# Fabrication and characterization of boric acid-crosslinked ethyl cellulose and polyvinyl alcohol films as potential drug release systems for topical drug delivery

**DOI:** 10.3906/kim-2008-23

**Published:** 2020-12-16

**Authors:** Nasrin ALIASGHARLOU, Farzin ASGHARI SANA, Saba KHOSHBAKHT, Pezhman ZOLFAGHARI, Hamed CHARKHIAN

**Affiliations:** 1 Department of Chemistry, Faculty of Science, Urmia University, Urmia Iran; 2 Department of Nanotechnology & Nanomedicine, Hacettepe University, Ankara Turkey; 3 Department of Pharmaceutics, School of Pharmacy, Urmia University of Medical Science, Urmia Iran; 4 Department of Chemical Engineering, Faculty of Chemical and Petroleum Engineering, University of Tabriz, Tabriz Iran; 5 Young Researchers Club, Urmia Branch, Islamic Azad University, Urmia Iran

**Keywords:** Drug delivery system, polymeric film, polyvinyl alcohol, ethyl cellulose, boric acid, erythromycin

## Abstract

In this study, boric acid (BA) is employed as a crosslinking agent to improve the characteristics of two commonly used polymeric films, ethyl cellulose (EC) and polyvinyl alcohol (PVA), for topical drug delivery applications. The developed films are characterized by FTIR spectroscopy and SEM analysis. The results show that the surfaces of the prepared films are even and transparent, except for the BA-modified EC sample. The initial cumulative release for erythromycin (EM) is found to be 0.30 and 0.36 mg/mL for EC and PVA films, which drops to 0.25 and 0.20 mg/mL after BA crosslinking, respectively, after 1 h at 25 °C. Further, the developed formulations are stable for 75 days. Also, the antibacterial activity of the developed formulations is investigated against *S. aureus* (ATCC® 25923™ and ATCC® 29213™). The obtained data confirm that the application of BA as the crosslinking agent extends the release of EM from EC and PVA polymeric films. The findings of this study suggest that BA-crosslinked EC and PVA films are promising carriers for controlled topical drug delivery applications.

## 1. Introduction

In recent years, drug delivery systems (DDSs) have been widely investigated by researchers owing to their numerous advantages, such as minimizing the side effects of drugs, better targeting, and cost-effectiveness [1]. Various sustained-release systems have been developed to adopt an effectively controlled drug release, including but not limited to particulate carriers, lipids, polymeric films, and gels [2–4].

Researchers have strived to develop pharmaceutical formulations based on polymeric films due to their drug-related properties, biocompatibility, and nontoxicity. Assorted natural and synthetic polymers have been used in film formation processes and classified into two major categories: hydrophilic and hydrophobic films [5,6].

Ethyl cellulose (EC), as a hydrophobic polysaccharide polymer, is a biodegradable and biocompatible polymer with unique properties such as high stability as well as heat, oxygen, and moisture resistance. Further, EC has desirable solubility in different organic solvents and is miscible with various water-soluble materials. It has excellent film-forming, filling, and adhesive capabilities, promoting its application in food, cosmetics, and pharmaceutical industries, especially as packaging material, coating, and encapsulating agents [7–9].Contrary to EC, polyvinyl alcohol (PVA) is a hydrophilic synthetic polymer with a linear and flexible structure. Being approved by Food and Drug Administration (FDA), PVA has widespread applications in cosmetics and pharmaceutical industries. It is known as a versatile carrier in sustained drug release systems in terms of various fibers, gels, and films due to its distinctive characteristics [10]. However, high moisture absorption and low mechanical strength hindered its application as a protective barrier [11–14].

The efficacy of polymeric films as DDSs is mainly determined by their stability, controlled, and selective drug release. Thus, the improvement of their properties is still a necessity to attain better results. Crosslinking is a promising method to enhance the structural and mechanical properties of polymers [15,16]. Boric acid (BA) is widely utilized in polymeric composites, owing to its ease of functionalizing and safety. It has been established as an eco-friendly material and can aid plant growth by acting as a nutrition source. Additionally, its therapeutic effects against a wide range of bacteria, yeast, and fungi are well proven [17–19]. It was also proven that boronic acid derivatives are selective inhibitors of the serine protease (β-lactamase family), while being cheaper than other alternatives. The incorporation of BA with antibiotics can offer a new platform to overcome antibiotic resistance, which is a major public health challenge [20,21].

This study aimed to develop erythromycin-loaded PVA and EC films as topical controlled-release drug delivery systems. Erythromycin (EM) is a hydrophobic macrolide antibiotic with a therapeutic effect against gram-positive and gram-negative bacteria via blocking protein synthesis in the ribosome of bacteria. However, EM contains a high quantity of alcohol for topical treatments, provoking skin irritations in patients [22,23]. Nevertheless, to the best of our knowledge, the effect of BA on the physicochemical properties of polymeric matrices has sparsely been investigated. The main purpose of this work was to examine the effect of BA on the in vitro drug release and the physicochemical properties of PVA- and EC-based films. Also, the possibility of synergistic antibacterial activity of BA in conjunction with EM in the developed films was explored.

## 2. Materials and methods

### 2.1. Materials

Erythromycin was purchased from Sepidaj Pharmaceutical, Iran. PVA (MW: 89000-91000 g/mol) and EC (Ethoxy groups, viscosity: 10 mPa.s) was obtained from Sigma-Aldrich, USA. Two standard strains of *S. aureus* , including *S. aureus* (ATCC® 25923™) and *S. aureus* (ATCC® 29213™), were purchased from KWICK-STICK Microbiologics, France. Mueller-Hinton Agar (MHA) and Mueller-Hinton Broth (MHB) were obtained from Becton Dickinson, France. The RPMI (Roswell Park Memorial Institute) 1640 W/L-Glutamine was obtained from Bio West, Germany. All other chemicals and reagents are analytical grade and used without purification.

### 2.2. Film preparation

EC films were prepared by dissolving 5% w/v of EC in an ethanol-water solution (80% w/w of ethanol). PVA films were fabricated by the addition of PVA (5% w/v) to deionized water. The developed solutions were heated up to 90 °C under mild stirring. To prepare the blended PVA-BA and EC-BA mixtures, the solutions were cooled down to reach a temperature of 45 °C, and 1% w/v BA was added to the resulted solutions under mild stirring. After the dissolution of BA, the pH was adjusted to 7 using a 1M NaOH. Afterward, EM (0.75% w/v) was added to the prepared gels and poured into Petri dishes. Finally, the developed films were dried in a vacuum chamber. Table 1 reports the composition of the developed films and their respective abbreviations, which will be utilized throughout this paper.

**Table 1:** Composition of the polymeric films.

Abbreviation	PVA (% w/v)	EC (% w/v)	BA (% w/v)	EM (% w/v)	DrugLoading (%)
P.f	5	–	–	–	–
E.f	–	5	–	–	–
PB.f	5	–	1	–	–
EB.f	–	5	1	–	–
P-EM.f	5	–	–	0.075	1.45
E-EM.f	–	5	–	0.075	1.5
PB-EM.f	5	–	1	0.075	1.22
EB-EM.f	–	5	1	0.075	1.26

### 2.3. Characterization

The morphological structures of the films were assessed using a scanning electron microscope (SEM; Zeiss Co., Germany) with an acceleration voltage of 20 kV. Fourier transform infrared spectrometer (FTIR; Perkin Elmer) was utilized to characterize the chemical structures of PVA, EC, and crosslinked films. The spectra were recorded in the range of 500 to 4000 cm ^-1^ with a resolution of 4 cm ^-1^ .

### 2.4. Swelling index and thickness

The effect of BA on the swelling behavior of the developed films was examined in water. Samples were prepared with a surface area of 100 × 100 mm, weighed, and then immersed in the aqueous medium. The weight of the dried sample was determined before and after the swelling process, while the surface wetness was removed using dry filter paper. The swelling index of each film was calculated using the following equation:

(1)swelling index(SI)=Wt-W0W0x100

Here *W* *t* is the weight of swollen film at time *t* , and *W* 0 corresponds to the dry film sample, respectively [24,25]. The film’s thickness (mm) was measured using a Palmer digital micrometer. The measurements were conducted at different points of each sample and the average value was reported [26].

### 2.5. In vitro drug release

The actual EM content was determined using the total immersion method [27]. In brief, a predetermined amount of each sample was dissolved in phosphate buffer solution (PBS, pH: 7.4) and placed in a shaker incubator (rotation speed of 100 rpm) at the temperatures of 25 and 32 °C. Eventually, a desired amount of samples was collected and replaced with the same volume of fresh PBS. The cumulative drug release was determined by UV-vis spectrophotometer (Cecil CE 7200, UK) at the wavelength of 202 nm. The following equation was applied to measure the EM Loading dose (%):

(2)Loading dose%=Drug content (mg)Nano fiber weight (mg)x100

### 2.6. Stability studies

The stability tests of EM-loaded films were carried out at 25 ºC (room temperature) and 4 ºC (fridge temperature) for 75 days. For this purpose, samples were collected to measure the drug content spectrophotometrically at the wavelength of 202 nm [28].

### 2.7. Zone of inhibition for antibacterial activities

Antibacterial activities of the prepared polymer-based formulations were evaluated against gram-positive bacteria *S. aureus* (ATCC® 25923™ and ATCC® 29213™) by measuring the inhibition zone around each film disc using disc diffusion methodology. MHA plates were seeded with 0.1 mL of bacteria suspensions with a bacterium count of 108 CFU/mL. Circular discs with a diameter of 6 mm were obtained from the films and placed on the MHA medium previously inoculated with the test bacteria. The agar plates were incubated at 35 °C for 24 h and the inhibition zones of the developed formulations were determined [29].

## 3. Results and discussions

### 3.1. Characterization

**Figure 1:**
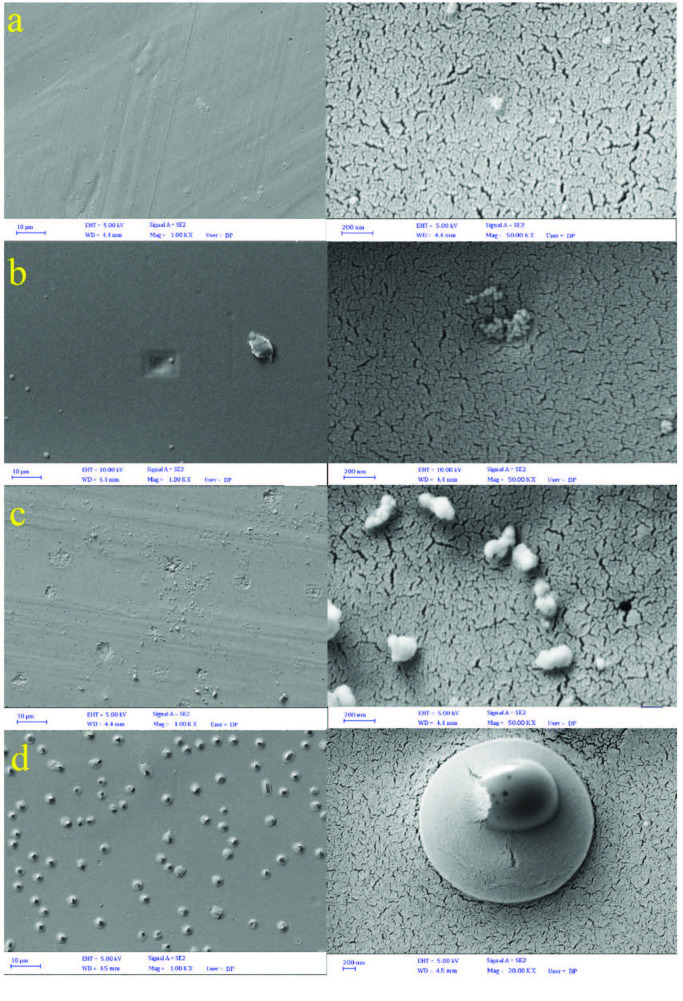
SEM images of the fabricated films; (a) P-EM.f, (b) PB-EM.f, (c) E-EM.f, and (d) EB-EM.f.

The SEM images of the EM-loaded blank (E-EM.f and P-EM.f) and crosslinked (EB-EM.f and PB-EM.f) films are shown in Figure 1. The P-EM.f sample displayed a smooth and even surface. Likewise, the E-EM.f micrographs revealed a slightly homogenous surface with some nanopores corresponding to the application of the mixture of water-ethanol as the solvent. Hjartstam et al. fabricated EC film by dissolving EC powder in various mixtures of water/ethanol compositions and reported that the film porosity increases with the elevation of water content [30]. The PB-EM.f film had a comparatively smooth surface texture, which showed that BA was scattered homogenously, improving the surface integrity. On the other hand, the presence of BA in the EB-EM.f film formed some circular bubbles instead of pores on the film’s surface. Also, the SEM micrographs indicated the presence of some insolubilized EM particles on the surface of the films.

**Figure 2:**
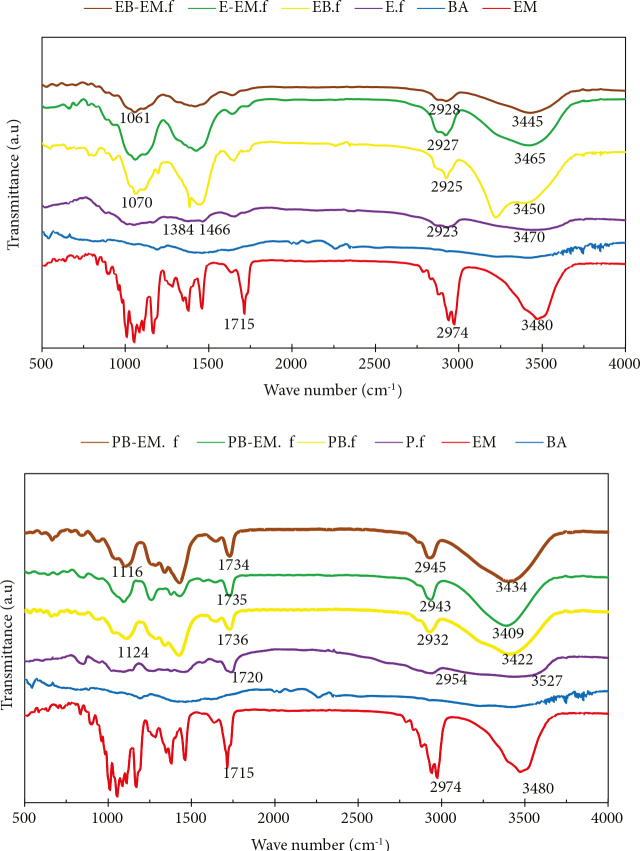
FTIR spectra of EM, BA, and the developed a) EC-based films; b) PVA-based films

Figure 2 presents the FTIR spectra of the fabricated films. For the P.f sample, an absorption peak was observed around the wavenumber of 3525 cm ^-1^ , which is ascribed to the vibrations of hydroxyl groups, while the peak at 1720 cm ^-1^ is attributed to the stretching of the C=O group. Also, the C–H bond vibrations resulted in a sharp band at 2954 cm ^-1^ [31]. The FTIR spectra curve of pure E.f sample demonstrated peaks at 3470, 2923, 1466, and 1384 cm ^-1^ corresponding to OH, CH, CH2, and CH3 bond stretching vibrations, respectively. The formulations containing boric acid exhibited a peak within the region of 1050–1150 cm ^-1^ , suggesting the formation of B-O-C bonds [32,33]. The FTIR spectra of EM indicated absorbances at 3480, 2974, and 1715 cm−1, corresponding to OH, CH, and C=O stretching vibrations, respectively. However, there was no discrete band in the blank and EM-loaded films (E-EM.F, P-EM.F, EB-EM.F, and PB-EM.F), which may be related to lack of chemical reaction between EM and the polymers [34].

### 3.2. Swelling index and thickness

**Figure 3:**
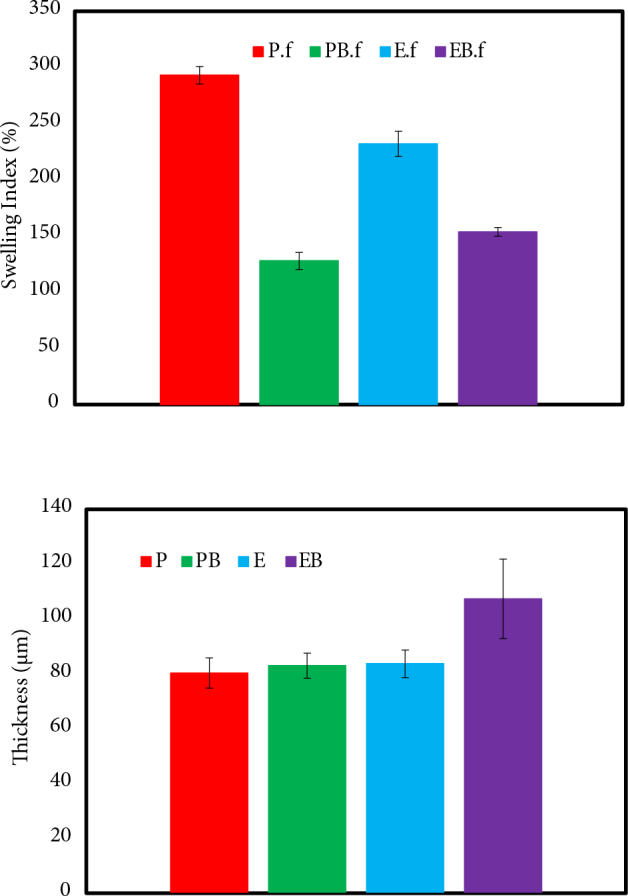
Swelling index (a) and Thickness (b) of the developed films

The swelling index (SI) of the developed films was measured at room temperature. The results depicted by Figure 3 revealed that the EB.f and PB.f samples have had a lower SI compared to E.f and P.f films, which can be related to the crosslinking and filling effect of BA [25]. Accordingly, BA increases the viscosity of the polymeric film and fills the pores of the polymeric films. As shown in Figure 3, the introduction of BA to the PVA film (PB.f) caused a more significant reduction in SI compared to BA-crosslinked EC samples (EB.f), which may be associated with the stronger crosslinking effect of BA with PVA.

As a drug delivery platform, the developed films should exhibit an even surface, which guarantees consistent mechanical properties and drug distribution. For this purpose, an equal amount of homogenous gels was poured into the Petri dishes located on an even surface to form the films. Figure 3 depicts the mean thickness values of the manufactured films varying from 80.33 to 107.33 mm. Also, insignificant standard deviation values verify the homogenous formulation of the films, except for the EB.f sample, which has a relatively diverse thickness distribution [7].

### 3.3. In vitro drug release

**Figure 4:**
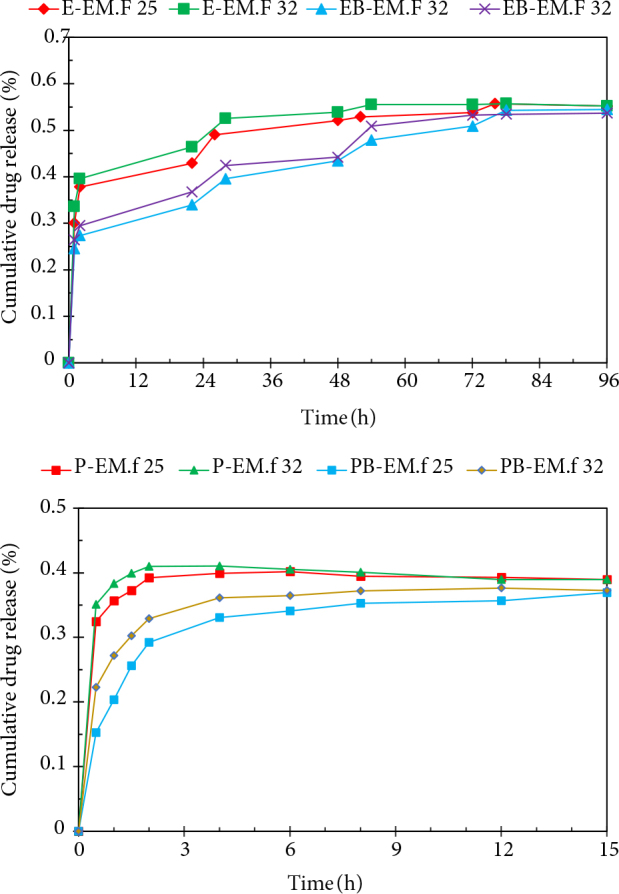
a) Release profiles of EM from E-EM.f and EB-EM.f films at 25 and 32 °C; b) EM Release profiles from P-EM.f and PB-EM.f films at 25 and 32 °C.

The EM loading quantities for the developed formulations are tabulated in Table 1. According to this table, drug loading decreased upon the addition of BA. Figure 4 demonstrates the in vitro EM release profile of all samples carried out in phosphate buffer at 25 °C (room temperature) and 32 °C (simulated skin temperature). The cumulative EM release was found to be 0.36, 0.20, 0.30, and 0.25 mg/mL in the first hour for P-EM.F, PB-EM.F, E-EM.F, and EB-EM.F formulations, respectively, at 25 °C. As observed, all formulations exhibited a rapid drug release in the first hours, followed by a sustained drug release path, which is desirable for the treatment of skin infections, considering the initial pathological load. P-EM.F and PB-EM.F samples released more than 0.35 mg/mL of the drug during the first and eighth hours, respectively, which was sustained with a slower rate after 15 h. However, E-EM.F and EB-EM.F samples reached this milestone at 2 and 22 h at the temperature 25 °C, respectively, promising a finely controlled drug release.

The initial burst EM release may result from the presence of drug particles on the surface of the films, which was confirmed by the SEM micrographs. Various parameters affect the drug diffusion from a polymeric matrix, including temperature, degradation of the polymeric matrix, and the solubility behavior of drug and polymer. In this research, the model drug (EM) was hydrophobic, retarding its release from the polymeric matrix with a slow-release path when compared to hydrophilic drugs. It was shown that EC-based drug formulations have a prolonged drug release profile due to the hydrophobic nature of the polymeric carrier, leading to a lower erosion rate of EC films in aqueous environments. As a result, the entrapped EM molecules in the EC polymeric matrix diffused slowly to the phosphate buffer medium. On the other hand, EM had a shorter release path using PVA carrier due to the fast dissolution of the polymer in aqueous solutions [35]. For all of the formulations, the EM release rate was higher at 32 °C compared to 25 °C [36]. This phenomena can be attributed to the lower stability of EM at higher temperatures [23,37].

The introduction of BA as the crosslinking agent formed hydrogen bonds, which were weakened at higher temperatures and led to a reduction of the crosslinking effect [38]. Further, PB-EM.f and EB-EM.f films freed EM at a slow rate in comparison to P-EM.f and E-EM.f formulations, respectively. BA could enhance the viscosity and surface tension of the system, delaying drug diffusion in the polymeric matrix. Also, BA lowered the dissolution rate and increased the water resistance of PVA [25]. Subrahmanyam et al. prepared theophylline tablets using guar gum and borax modified guar gum as a colon DDS. It has been reported that borax-crosslinked guar gum had a slower release path, explained by the enhancement of the viscosity [39]. In another study, Martinez et al. studied the effect of the glutaraldehyde, genipin, and disulfide as crosslinking agents on the in vitro drug release of protein polymers. The results revealed that crosslinked films exhibited a reduced initial burst release of rapamycin [40]. Various polymers can be used in film formulations as DDSs, depending on the release pattern required for the treatment of infections. The obtained drug release results indicate that the introduction of BA as the crosslinking agent successfully improved the sustained release of EM using both polymeric carriers.

Kown et al. employed EM-loaded PVA films as a controlled transdermal drug release system for acne treatment. In their study, they added polyethylene glycol600 and glycerol to the fabricated films to extend the EM release profile. The release of EM from blended polyethylene glycol/PVA showed an initial burst release with a sustained release for the next hours, which was similar to the drug release behavior of PB-EM.f sample [41].

### 3.4. Stability

**Figure 5:**
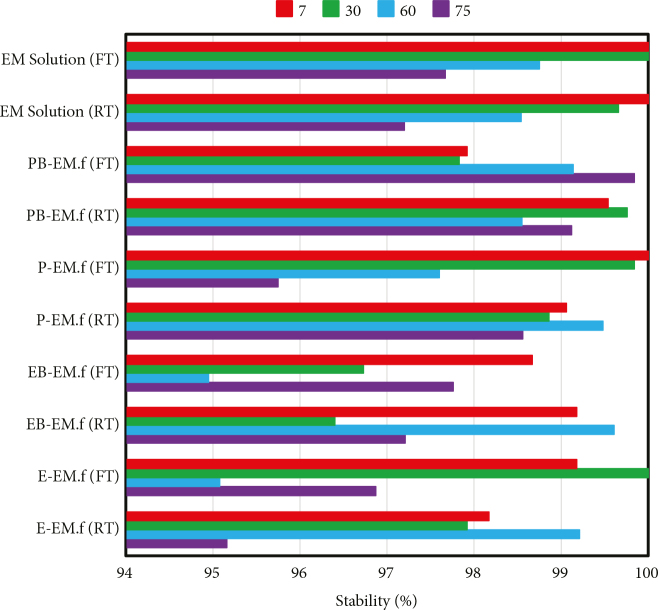
Stability tests of the fabricated formulation after 7, 30, 60, and 75 days.

The stability of EM in the developed EM-loaded films at room (RT, 25 °C) and fridge (FT, 4 °C) temperatures was assessed for 75 days. The aqueous drug solution was used as the control group and sealed to prevent light irradiation and drug degradation. After 75 days, the stability of the control sample was found to be 97.2 ± 2.12% and 97.67 ± 1.49% at RT and FT, respectively. As observed in Figure 5, the drug was stable over 75 days of storage in all formulations. Further, there was no difference in the EM stability of the drug-loaded films at both investigated temperatures, eliminating a low-temperature storage requirement.

However, it was found that borate ion accelerates the degradation of some drugs such as benzylpenicillin, carbenicillin, cefotaxime, atropine, hydrocortisone, indomethacin, methotrexate, oxytetracycline, minocycline, and 5-fluorouracil [42]. In the present study, it was shown that BA, as a borate compound, did not have any negative effect on the EM stability.

### 3.5. In vitro antibacterial assay

**Figure 6:**
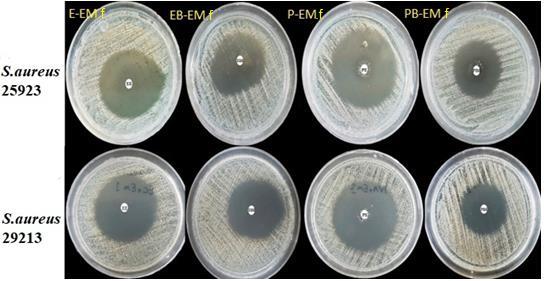
Inhibition zones of E-EM.f, EB-EM.f, P-EM.f and PB-EM.f samples against S. aureus ATCC25923 and S. aureus ATCC29213.

The antimicrobial activity of the prepared films was investigated against methicillin-susceptible *S. aureus* (ATCC _®_ 25923 _™_ ), and oxacillin sensitive *S. aureus* (ATCC _®_ 29213 _™_ ) by Kirby Bauer disc diffusion tests (Figure 6). As it was shown in Table 2, EB.f and PB.f formulations did not exhibit any antibacterial activity against (ATCC _®_ 25923 _™_ ) and (ATCC _®_ 29213 _™_ ). Boric acid as a crosslinking agent forms bonds with the polymers via hydroxyl groups. Also, it was demonstrated that an increase in the number of free B-OH groups directly improves the antibacterial effect [19,43,44]. However, in the present study, BA may be surrounded by PVA and EC molecules, which reduces the number of free B-OH groups, leading to the inhibition of the bacterial growth [45].

**Table 2:** Antibacterial activity of the developed formulations and standard antibiotic.

Strains	Sensitivity diameter (mm)						
	Samples						Standard antibiotic
	PB.f	P-EM.f	PB-EM.f	EB.f	E-EM.f	EB-EM.f	EM 15
S. aureus ATCC25923	0	30	27	0	30	32	30
S. aureus ATCC29213	0	38	22	0	38	30	33

Accordingly, E-EM.f and P-EM.f samples had similar antibacterial effects against (ATCC® 25,923™) and (ATCC® 29,213™), with inhibition zones of 30 and 38 mm, respectively. However, BA demonstrated synergistic antibacterial activity [46], noncrosslinked films expressing a larger inhibition zone compared to crosslinked formulations. Further, the EB-EM.f sample showed a greater inhibition zone against both bacteria in comparison with PB-EM.f. These may be related to the crosslinking effect of BA on the polymers, retarding EM diffusion. As shown in Figure 4, BA delayed EM diffusion from the polymeric matrix.

## 4. Conclusions

In the current study, BA-crosslinked EC and PVA films were developed for topical drug delivery. The effect of BA was examined on the morphological structure, physicochemical properties, drug release, stability, and antibacterial activity of EM-loaded EC and PVA films. The results showed good compatibility between EM particles and polymeric films. The drug was stable in all formulations after 75 days, offering exceptional drug stability, regardless of storage conditions. The introduction of BA to the PVA and EC films not only managed to improve their swelling behaviors but also extend the drug release path, reducing the frequency of dressing change. The results obtained from the present study suggest that BA-modified EC and PVA films can be considered as efficient choices for various real-world topical DDSs without the side effects of conventional drug formulations. Ultimately, BA, as a cost-effective β-lactamases inhibitor, can be applied in conjunction with other antibiotics for combating bacterial resistance, which requires further investigations.
